# Chemical Compensation of Mitochondrial Phospholipid Depletion in Yeast and Animal Models of Parkinson’s Disease

**DOI:** 10.1371/journal.pone.0164465

**Published:** 2016-10-13

**Authors:** Shaoxiao Wang, Siyuan Zhang, Chuan Xu, Addie Barron, Floyd Galiano, Dhaval Patel, Yong Joo Lee, Guy A. Caldwell, Kim A. Caldwell, Stephan N. Witt

**Affiliations:** 1 Department of Biochemistry and Molecular Biology, Louisiana State University Health Sciences Center, Shreveport, LA, United States of America; 2 Department of Biological Sciences, The University of Alabama, Tuscaloosa, AL, United States of America; 3 Department of Pharmacology, Toxicology and Neuroscience, Louisiana State University Health Sciences Center, Shreveport, LA, United States of America; Hertie Institute for Clinical Brain Research and German Center for Neurodegenerative Diseases, GERMANY

## Abstract

We have been investigating the role that phosphatidylethanolamine (PE) and phosphatidylcholine (PC) content plays in modulating the solubility of the Parkinson’s disease protein alpha-synuclein (α-syn) using *Saccharomyces cerevisiae* and *Caenorhabditis elegans*. One enzyme that synthesizes PE is the conserved enzyme phosphatidylserine decarboxylase (Psd1/yeast; *PSD-1*/worms), which is lodged in the inner mitochondrial membrane. We previously found that decreasing the level of PE due to knockdown of Psd1/*psd-1* affects the homeostasis of α-syn in vivo. In *S*. *cerevisiae*, the co-occurrence of low PE and α-syn in *psd1*Δ cells triggers mitochondrial defects, stress in the endoplasmic reticulum, misprocessing of glycosylphosphatidylinositol-anchored proteins, and a 3-fold increase in the level of α-syn. The goal of this study was to identify drugs that rescue this phenotype. We screened the Prestwick library of 1121 Food and Drug Administration-approved drugs using *psd1*Δ + α-syn cells and identified cyclosporin A, meclofenoxate hydrochloride, and sulfaphenazole as putative protective compounds. The protective activity of these drugs was corroborated using *C*. *elegans* in which α-syn is expressed specifically in the dopaminergic neurons, with *psd-1* depleted by RNAi. Worm populations were examined for dopaminergic neuron survival following *psd-1* knockdown. Exposure to cyclosporine, meclofenoxate, and sulfaphenazole significantly enhanced survival at day 7 in α-syn-expressing worm populations whereby 50–55% of the populations displayed normal neurons, compared to only 10–15% of untreated animals. We also found that all three drugs rescued worms expressing α-syn in dopaminergic neurons that were deficient in the phospholipid cardiolipin following cardiolipin synthase (*crls-1*) depletion by RNAi. We discuss how these drugs might block α-syn pathology in dopaminergic neurons.

## Introduction

Parkinson’s disease (PD) affects 1–2% of the population over 65 years of age and is the most common movement disorder. The disease is a consequence of the selective degeneration of dopaminergic neurons in a region of the mid-brain called the *substantia nigra* [1]. Loss of these neurons results in slowness of movement, rigidity, and postural instability. The affected neurons often display cytoplasmic inclusions called Lewy bodies, whose main component is the protein α-syn [2]. Post-translationally modified forms of α-syn or an accumulation of α-syn due to age-related declines in the protein degradation pathways likely cause sporadic cases of PD. Missense mutations in α-syn [3] or duplications/triplications [4] of the locus result in early-onset PD. In some individuals, if α-syn slowly accumulates over time, eventually toxic, oligomeric conformations may form and disrupt cell function, leading to cell death. The toxic conformations kill the host neurons and spread to healthy neighboring neurons.

Highly expressed in the brain, α-syn is also present in red blood cells, intestinal cells, liver cells, and melanocytes. α-syn, which has sequence similarity to lipid binding proteins [5], binds to membranes, vesicles, and even sequesters large numbers of lipid molecules to form nanoparticles [6, 7], consistent with it being a lipid carrier. α-syn has also been proposed to act in concert with soluble N-ethylmaleimide-sensitive factor attachment protein receptor (SNARE) proteins to facilitate synaptic vesicle fusion with the presynaptic membrane [8]. A wealth of evidence is consistent with α-syn changing its structure in a context-dependent manner. That is, α-syn is intrinsically disordered in solution [9] but upon binding to membranes it adopts a α-helical conformation [10]. If α-syn builds up in cells, then it self-associates into a myriad array of soluble protofibrils, some of which may be toxic [11]. α-syn can also form amyloid fibers. Preformed fibers of α-syn, when injected into healthy mice, cause a rapid neurodegenerative disease consistent with PD [12]. The molecular details as to how α-syn changes conformations, kills, and spreads are the subjects of intense investigations.

**Fig 1:**
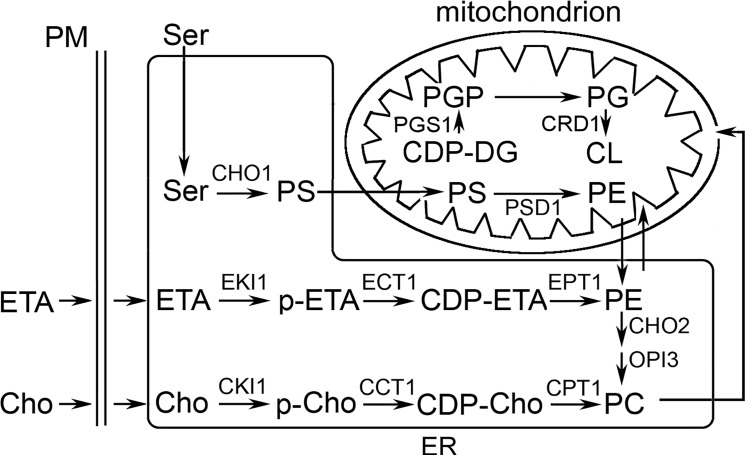
PE and CL synthesis in mitochondria and ER. CDP, cytidine diphosphate; Cho, choline; DG, diacylglycerol; ER, endoplasmic reticulum; ETA, ethanolamine; p-ETA/p-Cho, phosphorylated ETA/choline; PM, plasma membrane; PS, phosphatidylserine. Mitochondrial PE deficiency causes mitochondrial defects, ER and cell wall stress, misprocessing of glycosylphosphatidylinositol-anchored proteins, accumulation of α-syn. Cardiolipin deficiency causes defects in mitochondrial bioenergetics [23].

PE and its metabolites can decline in the brain with age [13–17]. α-syn is thought to slowly aggregate and form inclusions in neurons with age. In light of these phenomena, we hypothesized that decreasing the level of PE in cells would affect α-syn homeostasis, possibly leading to inclusion/foci formation. To this end, we used *S*. *cerevisiae* and *C*. *elegans* models of PD. The various pathways for the formation of PE and the enzymes that synthesize PE are conserved in yeast, worms, flies and mammals [18] (Fig 1). First, lodged in the inner membrane, the enzyme Psd1 converts phosphatidylserine to PE [19]. PE synthesized in the inner mitochondrial membrane can spread via mitochondrial-associated membranes to other cellular compartments [20, 21]. Second, the cytidine diphosphate (CDP)-ethanolamine (Kennedy) pathway consists of three enzymes that convert the metabolite ethanolamine into PE [22]; the last enzyme in this pathway is embedded in the membranes of the endoplasmic reticulum (ER). In some cells, Psd1 may synthesize most of the PE whereas in other cells the Kennedy pathway may synthesize the most of the PE.

Using yeast and worms, we showed that decreasing the level of PE by knocking down the gene coding for phosphatidylserine decarboxylase triggers mitochondrial defects, stress in the ER, misprocessing of glycosylphosphatidylinositol-anchored-anchored proteins, and a 3-fold increase in the level of α-syn [24]. Supplementation of yeast or worms with ethanolamine, which converts to PE via the CDP-ethanolamine pathway, abolished the extramitochondrial defects due to the co-occurrence of low PE (*psd1*Δ) and α-syn.

We were curious whether any Food and Drug Administration (FDA)-approved drugs would rescue cells with low PE and α-syn. High throughput screening identified three drugs—meclofenoxate hydrochloride (MFX), cyclosporine A (CsA), and sulfaphenazole (SUL)—that rescued the slow growth phenotype of *psd1*Δ cells expressing α-syn. The drugs were then further evaluated in a *C*. *elegans* model of α-syn-induced dopaminergic neurodegeneration.

## Results

### High Throughput Screen of Prestwick Library

**Fig 2:**
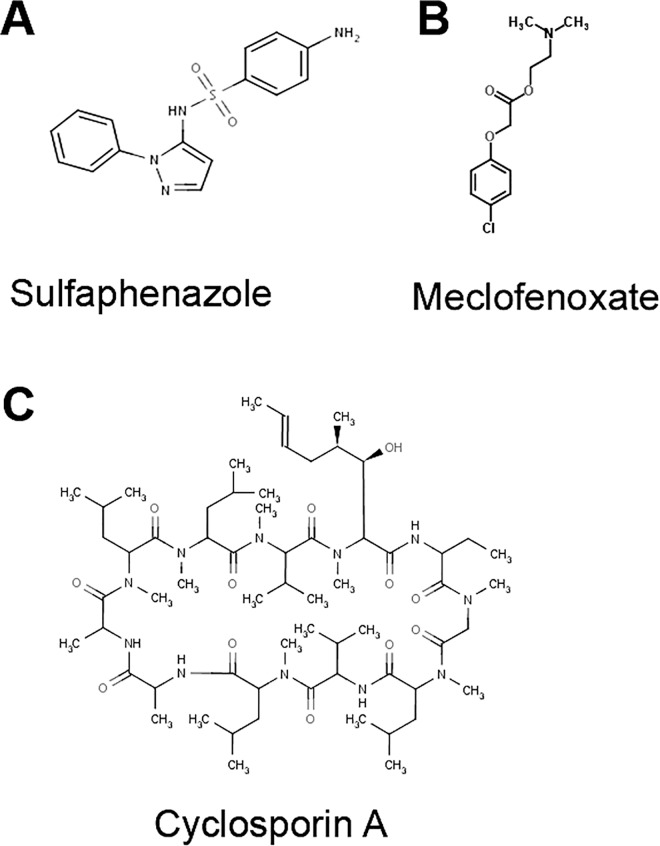
Structures of positive candidates identified from screen. (**A**) Sulfaphenazole (SUL), (**B**) meclofenoxate hydrochloride (MFX) and (**C**) cyclosporine A (CsA).

A high throughput screen of the Prestwick library of 1121 FDA-approved drugs was conducted to identify drugs that rescue the slow growth phenotype of *psd1*Δ yeast cells expressing human wild-type α-syn under the control of the Gal1 promoter. Ethanolamine was a positive control. Two hits—MFX and SUL—were identified that rescued the growth defect almost as well as ethanolamine, whereas CsA was one of the weaker hits (S1 Fig; S1–S4 Tables). These three drugs (Fig 2) of the highest purity were purchased from Sigma and retested in yeast.

**Fig 3:**
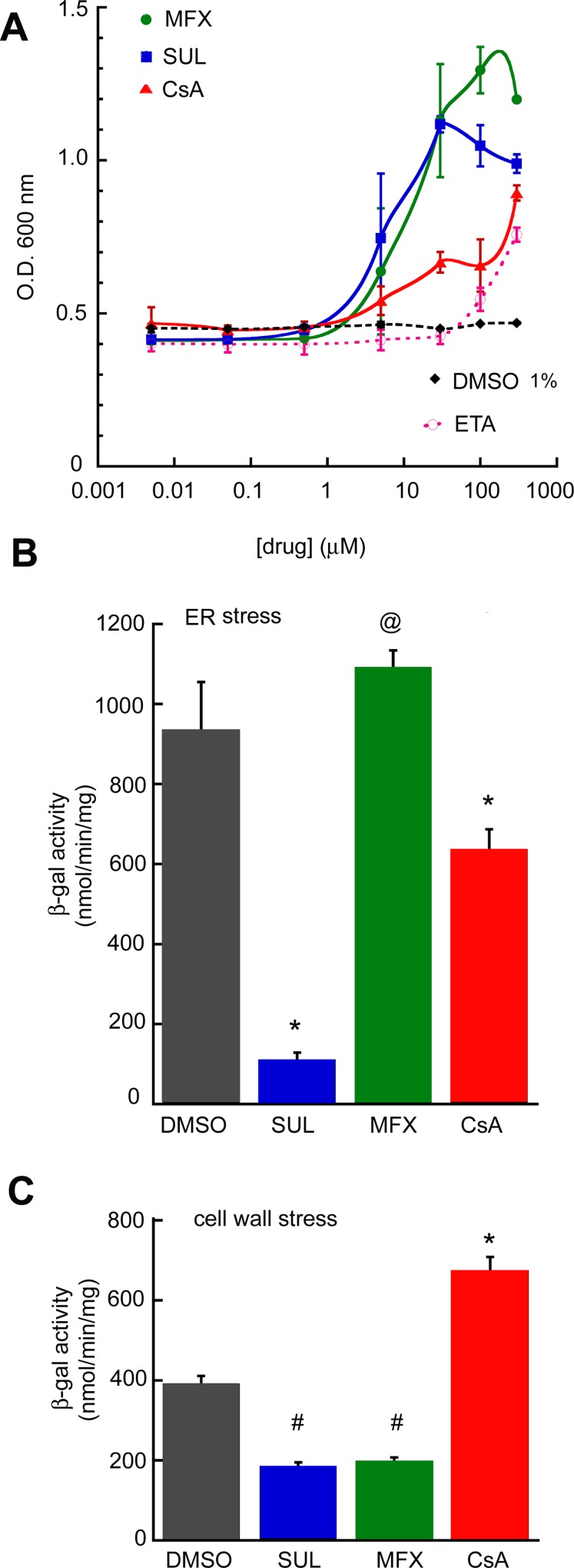
Activity of the candidate drugs in yeast. (**A**) Dose-response: *psd1*Δ cells expressing α-syn were diluted to O.D. = 0.4, drugs were added, and the absorbance was measured (OD 600) at 24 h at 30°C. For MFX, CsA, and SUL values for 5, 30, and 100 μM are means ± SD from two independent experiments (two replicates for each concentration); for 0.005–0.5 and 300 μM drug, values are from one experiment (two replicates for each concentration). For ethanolamine, values are from two independent experiments (each in triplicate). (◆), *psd1*Δ + α-syn + DMSO (1% v/v). (**B**) ER stress assay. β-gal activity of cells transformed with pAG425–α-syn plasmid and pMCZ-Y plasmid (UPRE–*lacZ*) induced for 8 h in the presence of the indicated drug, cells were lysed, and LacZ activity was measured. ^@^*p* = 0.02, *p ≤ 0.0001 (compared to DMSO) determined using one-way ANOVA, Dunnett post hoc test. (**C**) Cell wall stress assay. β-galactosidase activity of cells transformed with pAG425/pAG425-α-syn and 1366 plasmids, induced for 8 h with indicated drug, cells were lysed, and LacZ activity was measured. ^#^*p* = 0.001–0.002, *p < 0.0001 (compared to DMSO) determined using one-way ANOVA, Dunnett post hoc test. (**B**), (**C**) All values are means ± SD from two independent experiments (with two independent replicates for each drug per experiment). Concentrations of drugs: DMSO, 1% v/v, SUL, 30 μM, MFX, 30 μM, CsA 100 μM. The DMSO concentration in the cultures was 1% v/v.

To estimate dose-response relationships, each drug was retested in a growth assay in a liquid medium over a range of concentrations in *psd1*Δ/α-syn cells. The dose-response curves showed half-maximal responses of 10 μM for MFX and SUL and 100 μM for CsA (Fig 3A).

We have previously shown that *psd1*Δ cells expressing α-syn display stress in the ER and cell wall. Therefore, we tested each of the three drug candidates for their ability to inhibit ER stress using yeast carrying the reporter plasmid pMCZ-Y [24]. After culturing *psd1*Δ/α-syn cells for 8 h in inducing medium with the indicated drug cells were lysed and the LacZ activity, which is proportional to ER stress, was measured in the clarified lysate. SUL decreased ER stress by 88% compared to the control (DMSO) (p<0.0001) (Fig 3B), whereas CsA decreased ER stress by 32% (p = 0.0001) and MFX slightly increased ER stress (p = 0.02).

The three drug candidates were also tested for their ability to inhibit cell wall stress using a reporter plasmid in which the bacterial *lac*Z gene is controlled by the Rlm1-regulated promoter of PRM5 (*PPRM5*::*lacZ*) [25]. Prm5 is induced in response to activation of the cell wall integrity pathway. After culturing *psd1*Δ/α-syn cells for 8 h in inducing medium with the various drugs cells were lysed and the LacZ activity, which is proportional to cell wall stress, was measured. SUL and MFX each decreased cell wall stress by ~50% compared to control (DMSO) (0.001 ≤ p ≤ 0.002) (Fig 3C). CsA increased cell wall stress compared to the control (p<0.0001). The three drugs appear to affect different pathways in *psd1*Δ cells (Table 1).

**Table 1:** Phenotypes of yeast and worms expressing α-syn after treatment with candidate drugs.

	Yeast—α-syn/low PE (*psd1*Δ)			Worm—α-syn/low PE or CL
Drug	Growth	ER stress	Cell wall stress	DA neuron loss
DMSO	negligible	yes	yes	yes
MFX	strong rescue	slightly enhance	rescue	rescue
SUL	strong rescue	strong rescue	rescue	rescue
CsA	weak rescue	weak rescue	enhance	rescue

**Fig 4:**
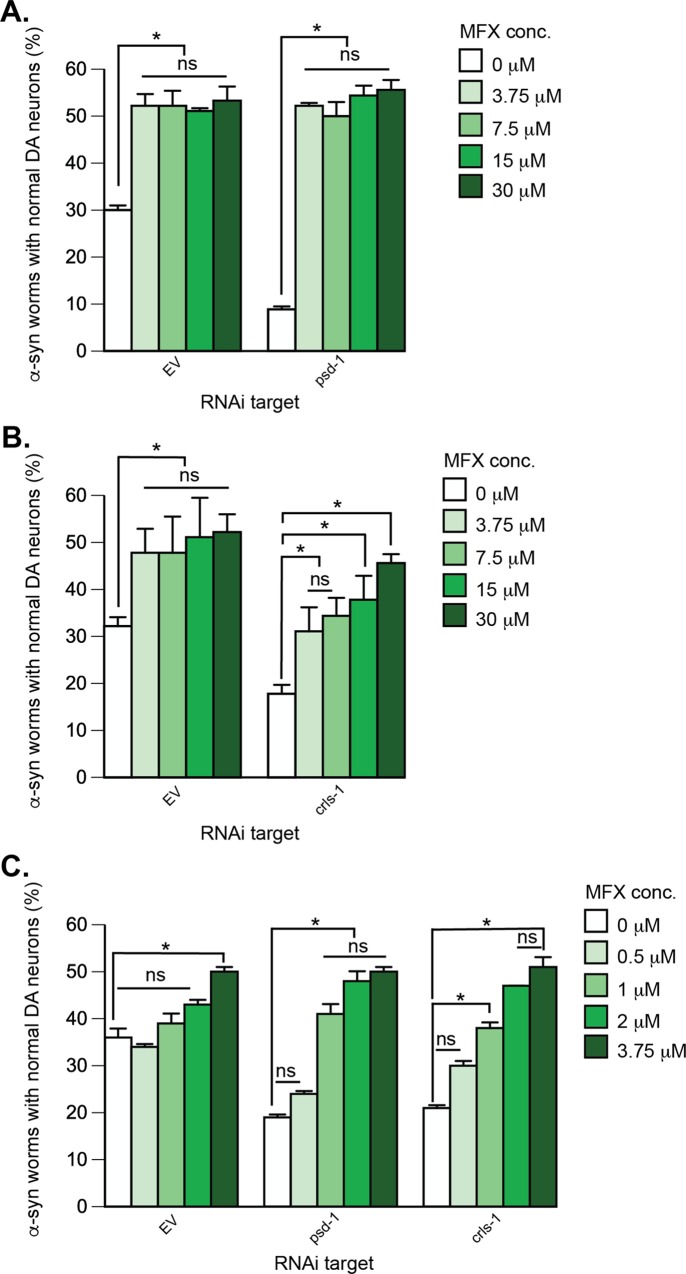
α-syn-induced dopaminergic neurodegeneration in *C*. *elegans* is rescued by MFX. Treatment of α-syn-expressing dopaminergic neurons with *psd-1* or *crls-1* RNAi, which causes enhanced neurodegeneration compared to α-syn alone, is also rescued by MFX (**A**—**C**). Graphical representation of *C*. *elegans* strain UA196 [*sid-1(pk3321)*; P_*dat-1*_::α-syn, P_*dat-1*_::GFP; P_*dat-1*_::*sid-1*, P_*myo-2*_::mCherry] following *psd-1* (**A, C**) or *crls-1* (**B, C**) knockdown. For RNAi experimental conditions, synchronized *C*. *elegans* were analyzed at day 7 post-hatching. RNAi bacteria, which do not express an RNAi clone (EV), were used as a negative control. A worm was scored as normal when it had a full complement of six anterior dopaminergic neurons. Data are reported as the mean ± SD, n = 90 worms. *p < 0.05, two-way ANOVA. (**A**) *C*. *elegans* fed with EV or *psd-1* dsRNA were treated with MFX (0, 3.75, 7.5, 15, 30 μM dissolved in 0.1% v/v DMSO). (**B**) The same MFX concentrations were analyzed following dopaminergic neuron-specific EV or *crls-1* knockdown. (**C**) *C*. *elegans* fed with EV, *psd-1*, *or crls-1* dsRNA were treated with MFX (0, 0.5, 1, 2, 3.75 μM dissolved in 0.1% v/v DMSO).

**Fig 5:**
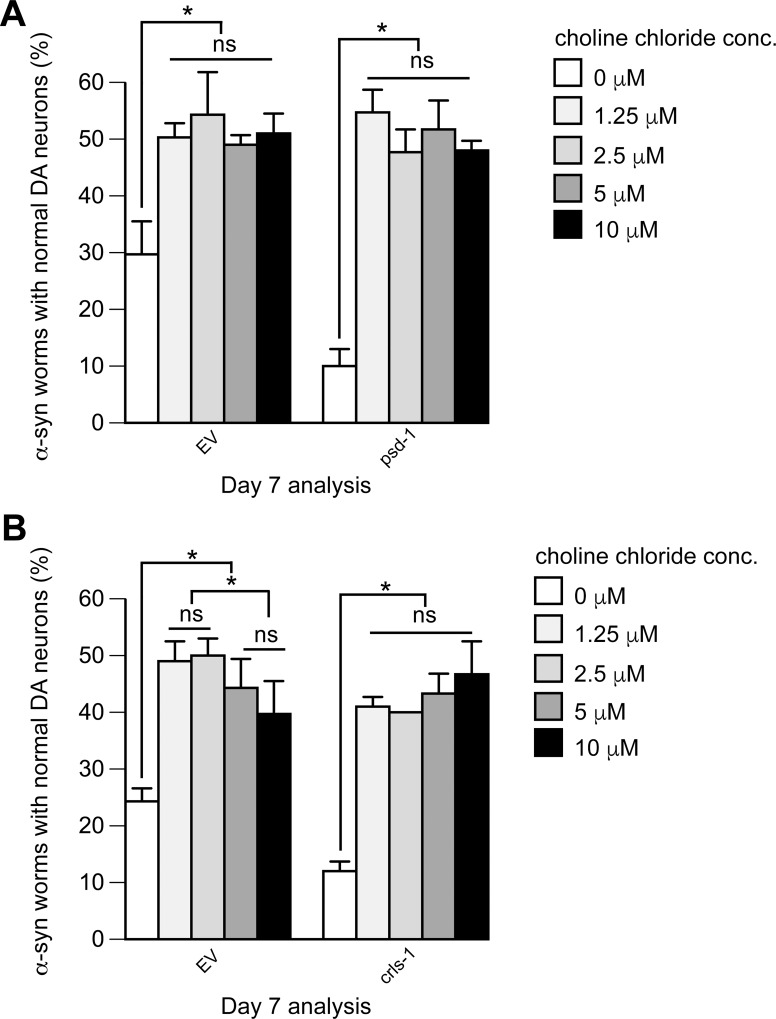
α-syn-induced dopaminergic neurodegeneration in *C*. *elegans* is rescued by choline chloride. Treatment of α-syn-expressing dopaminergic neurons with *psd-1* or *crls-1* RNAi, which causes enhanced neurodegeneration compared to α-syn alone, is also rescued by choline chloride (**A**, **B**). Graphical representation of *C*. *elegans* strain UA196 [*sid-1(pk3321);* P_*dat-1*_::α-syn, P_*dat-1*_::GFP; P_*dat-1*_::*sid-1*, P_*myo-2*_::mCherry] following *psd-1* (**A**) or *crls-1* (**B**) knockdown. For both RNAi experimental conditions, synchronized *C*. *elegans* were analyzed at day 7 post-hatching. RNAi bacteria, which do not express an RNAi clone (EV), were used as a negative control. A worm was scored as normal when it had a full complement of six anterior dopaminergic neurons. Data are reported as the mean ± SD, n = 90 worms. *p < 0.05, two-way ANOVA. (**A**) *C*. *elegans* fed with EV or *psd-1* dsRNA were treated with choline chloride (0, 1.25, 2.5, 5, 10 μM dissolved in water). (**B**) The same choline concentrations were analyzed following dopaminergic neuron-specific EV or *crls-1* knockdown.

**Fig 6:**
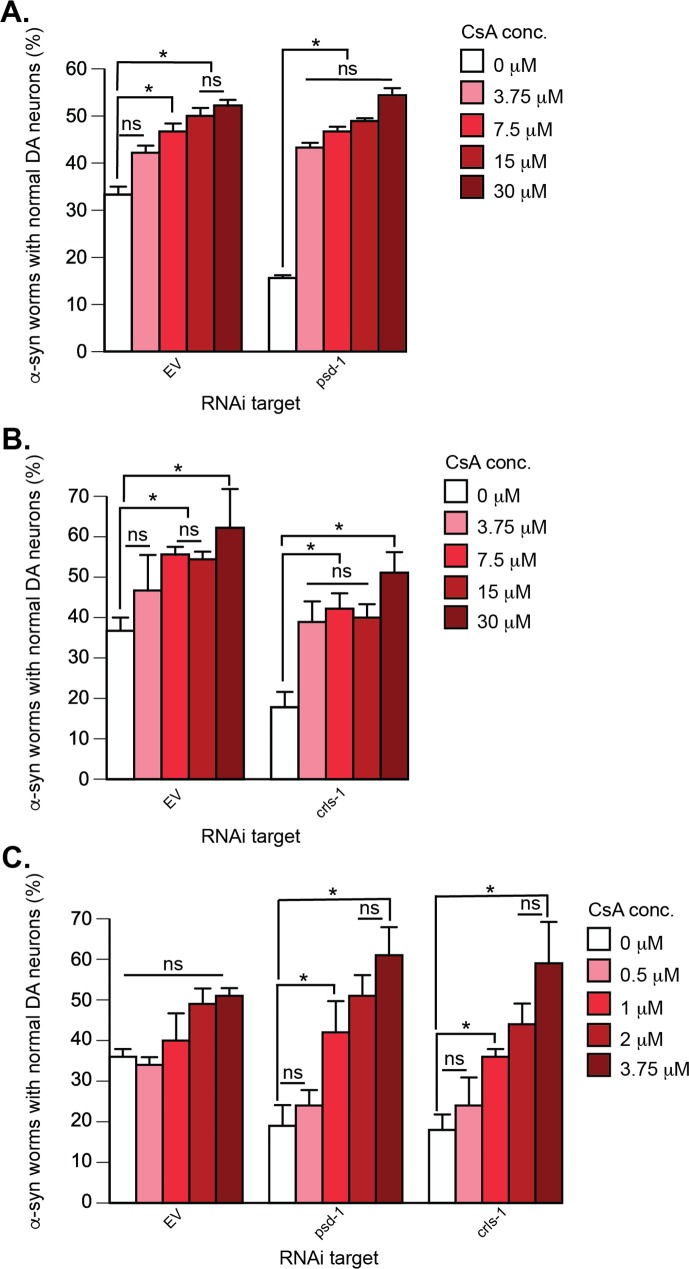
α-syn-induced dopaminergic neurodegeneration in *C*. *elegans* is rescued by CsA. Treatment of α-syn-expressing dopaminergic neurons with *psd-1* or *crls-1* RNAi, which causes enhanced neurodegeneration compared to α-syn alone, is also rescued by CsA. (**A**—**C**). Graphical representation of *C*. *elegans* strain UA196 [*sid-1(pk3321)*; P_*dat-1*_::α-syn, P_*dat-1*_::GFP; P_*dat-1*_::*sid-1*, P_*myo-2*_::mCherry] following *psd-1* (**A, C**) or *crls-1* (**B, C**) knockdown. For both RNAi experimental conditions, synchronized *C*. *elegans* were analyzed at day 7 post-hatching. RNAi bacteria, which do not express an RNAi clone (EV), were used as a negative control. A worm was scored as normal when it had a full complement of six anterior dopaminergic neurons. Data are reported as the mean ± SD, n = 90 worms. *p < 0.05, two-way ANOVA. (**A**) *C*. *elegans* fed with EV or *psd-1* dsRNA treated with CsA (0, 3.75, 7.5, 15, 30 μM dissolved in 0.1% v/v DMSO). (**B**) The same CsA concentrations were analyzed following dopaminergic neuron-specific EV or *crls-1* knockdown. **(C)** *C*. *elegans* EV, *psd-1*, or *crls-1* dsRNA were treated with CsA (0, 0.5, 1, 2, 3.75 μM dissolved in 0.1 v/v DMS0).

**Fig 7:**
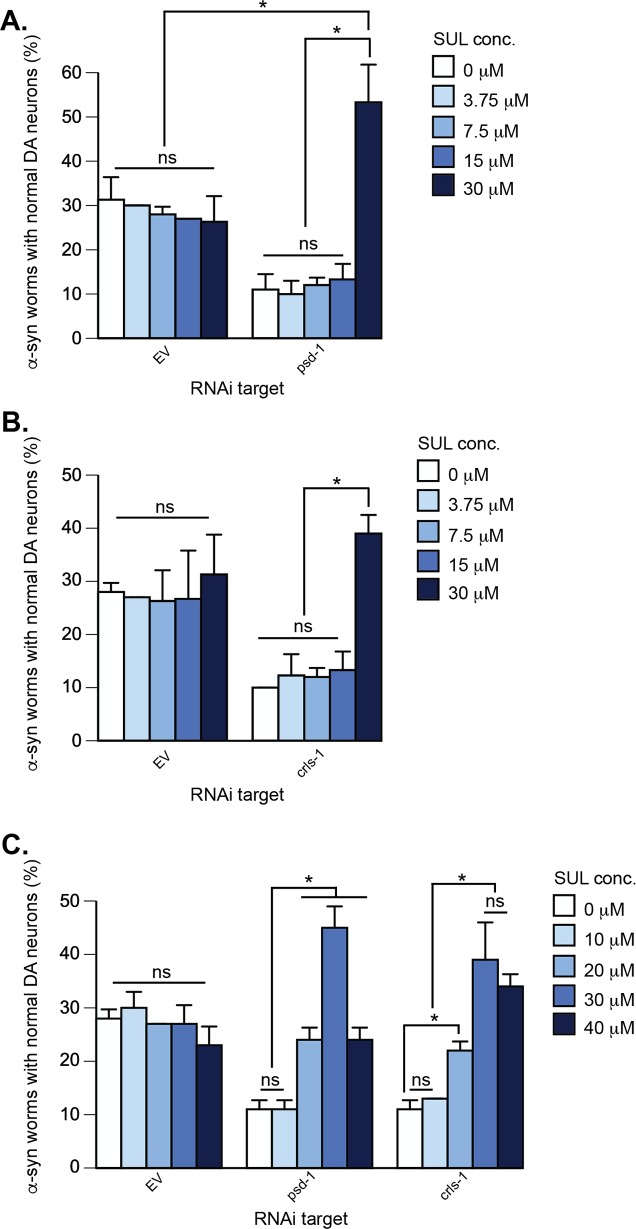
Depletion of *psd-1* or *crls-1* in α-syn-expressing dopaminergic neurons enhances neurodegeneration; this neurodegeneration is rescued by 30 μM SUL. Additionally, when dopaminergic neurons are depleted for *psd-1* and treated with 30 μM SUL they display enhanced protection beyond wild-type levels (**A**—**C**). Graphical representation of *C*. *elegans* strain UA196 [*sid-1(pk3321)*; P_*dat-1*_::α-syn, P_*dat-1*_::GFP; P_*dat-1*_::*sid-1*, P_*myo-2*_::mCherry] following *psd-1* (**A, C**) or *crls-1* (**B, C**) knockdown. For both RNAi experimental conditions, synchronized *C*. *elegans* were analyzed at day 7 post-hatching. RNAi bacteria, which do not express an RNAi clone (EV), were used as a negative control. A worm was scored as normal when it had a full complement of six anterior dopaminergic neurons. Data are reported as the mean ± SD, n = 90 worms. *p < 0.0005, two-way ANOVA. (**A**) *C*. *elegans* fed with EV or *psd-1* dsRNA were treated with SUL was treated (0, 3.75, 7.5, 15, 30 μM dissolved in 0.1% v/v DMSO). (**B**) The same SUL concentration range was tested following dopaminergic neuron-specific EV or *crls-1* knockdown. (**C**) *C*. *elegans* fed EV, *psd-1*, or *crls-1* dsRNA were treated with SUL (0, 10, 20, 30, 40 μM dissolved in 0.1% DMS0).

Tabulation of results from Figs 3–7.

### Corroboration Using a *C*. *elegans* Neurodegeneration Model

To further investigate the findings from yeast, we tested the drugs in a *C*. *elegans* neurodegeneration model where expression of wild-type (non-mutated) human α-syn cDNA under control of a dopamine transporter-specific promoter [P_*dat-1*_::α-syn *+* P_*dat-1*_::GFP] results in progressive, dose-dependent neurodegeneration [26, 27]. To enable depletion of neuronal genes using RNAi (RNA interference)-mediated silencing, the dopaminergic neuron-sensitive RNAi strain of *C*. *elegans* (UA196 [*sid-1(pk3321);* P*dat-1*::α-syn, P*dat-1*::GFP; P*dat-1*::*sid-1*, P*myo-2*::mCherry]) was used [28]. We have shown that RNAi silencing of *psd-1* expression enhances the toxicity of α-syn in the dopaminergic neurons [24]. Drugs were tested in UA196 worms with EV (empty vector) RNAi or *psd-1* knocked down by RNAi.

To expand our knowledge of the effects of lipid deficiencies in neurons, we asked how RNAi-depletion of cardiolipin synthase (*crls-1*) affects the survival of nematode dopaminergic neurons expressing α-syn. The phospholipid cardiolipin (CL) is critical for the proper functioning of the mitochondrial respiratory complexes and it predominantly localizes to the inner mitochondrial membrane. PE and CL are cone-shaped phospholipids that tend to impart negative curvature to bilayers [29]. Each of these phospholipids forms hexagonal phases and are considered “nonbilayer forming lipids”. Because PE and CL have similar biophysical properties and overlapping functions [30], we hypothesized that drugs that rescue cells with α-syn and low PE would also rescue those with α-syn and low or no CL. Drugs were also tested in UA196 worms with EV RNAi or *crls-1* RNAi.

Using the dopaminergic-specific α-syn RNAi strain, we depleted *psd-1* or *crls-1* by RNAi and analyzed dopaminergic neurodegeneration in worm populations compared to EV (negative control) RNAi. The solvent control treatments (DMSO or water) did not significantly interfere with the RNAi conditions. As described previously [24], at day 7 there was a significant difference between α-syn-expressing worms treated with EV + DMSO, where about 30% of the population displayed a full complement of dopaminergic neurons and α-syn-expressing worms treated with *psd-1* RNAi + DMSO, where about ~10%–15% of the population retained a full complement of neurons (Figs 4A–7A; *p* < 0.05; two-way ANOVA). Depleting *crls-1* in a DMSO background also results in ~10–15% of the population preserving their dopaminergic neurons (Figs 4B–7B; *p* < 0.05; two-way ANOVA).

### MFX, CsA, SUL, and Choline Protect Worm Dopaminergic Neurons that Express α-syn and with *psd-1* or *crls-1* Depleted by RNAi from Degeneration

**MFX.** Based on the results from yeast, we initially tested MFX at four doses ranging from 3.75 to 30 μM). MFX at 3.75 μM protected against dopaminergic cell loss in *psd-1* dsRNA and EV RNAi worms (Fig 4A). The rescue by MFX on *psd-1* depleted α-syn transgenic worms went from 9% at baseline treatment to ~53% at all concentrations tested (*p* < 0.0001, two-way ANOVA). Similarly, MFX increased the population of EV RNAi worms that displayed the full complement of neurons from 30% to 50% at all concentrations tested (*p* < 0.05). MFX also increased the percentage of α-syn expressing *crls-1* worms with a full complement of neurons in a dose-dependent fashion from 20 to 50% (*p* < 0.05, two-way ANOVA), with a half-maximal response at 8 μM MFX (Fig 4B). Given that the activity of MFX was detected at the lowest dose tested (3.75 μM), we proceeded to examine additional decreasing concentrations (Fig 4C) in both *psd-1* and *crls-1* knockdown worms. These latter studies revealed that concentrations 1 μM and above will rescue DA neurodegeneration in *psd-1* and *crls-1* depleted animals.

MFX is an ester of 4-chlorophenoxyacetic acid and dimethylethanolamine (DMAE). MFX is thought to hydrolyze into these two components inside cells [31]. Because DMAE (HO-CH_2_-N(CH_3_)_2_) and choline (HO-CH_2_-N^+^(CH_3_)_3_), which is an essential nutrient, differ by only a single methyl group, we also tested choline.

**Choline.** Choline at 1.25 μM protected against α-syn-induced dopaminergic cell loss in worms treated with *psd-1* dsRNA, *crls-1* dsRNA, and EV RNAi (Fig 5A and 5B). For example, after 7 days, there was a significant difference between α-syn-expressing worms with either *psd-1* or *crls-1* depleted, where only 10% of the population displayed a full complement of neurons, but in α-syn–expressing worms with *psd-1 or crls-1*–depleted treated with 1.25 μM choline, 50% or 40% of the respective populations displayed a full complement of dopaminergic neurons (*p* < 0.05, two-way ANOVA). Notably, after 7 days the population of α-syn expressing EV worms that retained a full complement of neurons was 25–30%, whereas the population of the same worms treated with choline (1.25 μM) increased to 50% (p< 0.05) (Fig 5A and 5B, left panels). For the various strains, increasing the dose above 1.25 μM protected no further, suggesting that the response is saturated at this concentration and that the dose for the half maximal response is less than 1 μM.

**CsA.** CsA protected against dopaminergic cell loss in *psd-1*, *crls-1*, and EV RNAi worms (Fig 6A and 6B). CsA treatment of α-syn expressing worms increased the number of worms with a full complement of dopaminergic neurons from 17% to 45% and 50% for worms with *crls-1* and *psd-1* knocked down, respectively. Full protection occurred in each case at the initial lowest dose of CsA tested, which was 3.75 μM (Fig 6A and 6B), suggesting that the half-maximal concentration of CsA is less than 3.75 μM. CsA treatment of α-syn expressing EV control worms exhibited a dose-response curve with a half-maximal CsA concentration of 8 μM. Therefore, we proceeded to examine additional lower concentrations (0.5–2 μM) (Fig 6C). These studies revealed that that concentrations 1 μM and above will rescue DA neurodegeneration in *psd-1* and *crls-1* depleted animals.

**SUL.** α-syn-expressing worms were less sensitive to SUL than the other drugs. SUL rescued the enhanced neurodegeneration in *psd-1* and *crls-1* depleted worms only at the highest initial concentration tested (30 μM). For example, SUL at 30 μM boosted the population of worms with a full complement of neurons from 11%/ 10% to 53%/ 40% in the *psd-1/crls-1* -depleted, α-syn-expressing worms (Fig 7A and 7B) (*p* < 0.0005, two-way ANOVA). Unlike the other drugs, SUL failed to protect the EV control worms from progressive α-syn-dependent neuron loss. To potentially discern a more detailed response profile for SUL, we conducted another set of trials using a broader range of concentrations (Fig 7C). Nevertheless, this subsequent analysis revealed that 30 μM is the optimal dosage yielding neuroprotection when *psd-1* or *crls-1* are knocked down by RNAi.

### α-Syn Expression Is Not Altered following Exposure to MFX, CsA, SUL or Choline in *C*. *elegans*

**Fig 8:**
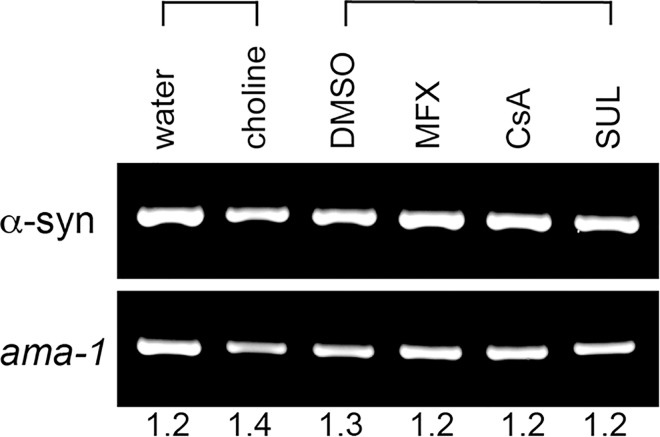
Choline, MFX, CsA, and SUL exposure do not affect α-syn expression. Stained agarose gel image depicting the products of semi-quantitative RT-PCR reactions following *C*. *elegans* strain UA196 [*sid-1*(pk3321); P_*dat-1*_::α-syn, P_*dat-1*_::GFP; P_*dat-1*_::*sid-1*, P_*myo-2*_:: mCherry] exposure to chemicals or solvents. Worms were exposed to the following treatments: 10 μM choline chloride (in ddH_2_O solvent), 30 μM MFX, 30 μM CsA, 30 μM SUL (all in 0.1% DMSO solvent). The products of RT-PCR are shown for α-syn primers (top panel) and *ama-1* loading control primers (bottom panel). Equal amounts of PCR product were loaded in each lane. The normalized intensity values for the various drug treatments and solvent controls are shown below the blot images.

We were curious to know if the neuroprotection from α-syn in *C*. *elegans* was due to lowered α-syn gene expression. We treated α-syn-expressing worms with the most effective rescuing concentration of each drug and then worms were harvested at day 7 post-hatching for α-syn mRNA analysis. This was also the day used for DA neurodegeneration analysis. We determined that α-syn expression is unchanged among various drug treatments and solvent controls (Fig 8). Thus, the DA neuroprotection observed is not altering α-syn expression levels.

## Discussion

We identified three drugs that compensate for mitochondrial phospholipid depletion in yeast and animal models of Parkinson’s disease. CsA and MFX have been previously reported to protect against neurodegenerative phenotypes in various models [32–35], whereas this is the first report that SUL protects against α-syn-induced DA neuron loss. A novel aspect of our study is that each of the three drug candidates partially rescues cells from the severe condition of the co-occurrence of α-syn and low PE (*psd1*Δ/*psd-1*) or low CL (*crls-1*) (Table 1). None of the drugs affected the expression of α-syn (Fig 8). Guided by reports on MFX, CsA, and SUL, we discuss below how these drugs might function to compensate for mitochondrial phospholipid depletion in our PD models.

**MFX.** MFX protects nematode DA neurons with or without lipid depletion from age- and α-syn-associated cell death (Fig 4). MFX (a.k.a. centrophenoxine) is a nootropic drug that is marketed as a memory enhancer. This drug, which readily crosses the blood-brain barrier, has been reported to inhibit enzymes involved in PC biosynthesis [36, 37], increase acetylcholine, scavenges radicals [38], and ameliorate rotenone-induced motor dysfunction in rodents [34, 35]. MFX rapidly hydrolyzes into 4-chlorophenoxyacetic acid and dimethylethanolamine (DMAE) at neutral pH, and DMAE is considered to be the active product because of its ability to scavenge hydroxyl radicals [39]. DMAE can also couple with diacylglycerol to yield phosphatidyl-DMAE, which incorporates into membranes and scavenges radicals [31]. Phosphatidyl-DMAE can also be methylated to PC. We suggest that MFX protects nematode DA neurons with or without lipid depletion from age- and α-syn-associated cell death by scavenging radicals or increasing the level of PC.

**Choline.** Choline partially ameliorates the synthetic toxicity of α-syn and the mitochondrial depletion of PE or CL; choline also partially ameliorates α-syn-induced neuron loss in worms (EV) without depletion of mitochondrial lipids (Fig 5). Feeding choline to worms should increase the level of PC by stimulating the CDP-phosphatidylcholine pathway (Fig 1). PC synthesized in the Kennedy pathway helps maintain α-syn in a soluble non-toxic state [24], and as shown here it rescues mitochondrial PE or PC depletion. Given that the CL and PC synthetic pathways do not intersect, how does choline rescue mitochondrial CL deficiency? First, although PC comprises 50% of the mitochondrial membranes, little is known about its role in mitochondrial protein biogenesis or stability. Second, uncharacterized homeostatic mechanisms exist that maintain the proper ratio of bilayer-forming lipids like PC to non-bilayer-forming lipids like CL and PE. This is evidenced by two recent discoveries using the *psd1*Δ yeast cells [21]. The level of PE decreases in *psd1*Δ cells compared to wild-type cells, as expected, whereas PC unexpectedly and by an unknown mechanism increases. This implies that PC compensates for low PE. Curiously, ethanolamine supplementation to *psd1*Δ cells increases the level of CL. We suggest that supplemental choline increases PC, and that PC compensates for low CL by the same or similar homeostatic mechanism by which supplemental ethanolamine increase CL.

**CsA.** CsA protected nematode dopamine neurons with or without lipid depletion from progressive α-syn-associated cell death (Fig 6). CsA is a powerful immunosuppressant that prevents T-cell activation by inhibiting Ca^++^-signaling [40]. CsA has a secondary activity in many types of cells, i.e., CsA-cyclophilin D complexes inhibit the mitochondrial permeability transition pore (mPTP) [41]. The mPTP is a non-specific pore that forms in the inner mitochondrial membranes in response to high matrix Ca^++^, arachidonic acid [42], ceramide [43], inorganic phosphate, and many other factors. Pore opening is reversible, although prolonged opening of the mPTP results in the collapse of the proton motive force, release of matrix NADH, and leakage of cytochrome *c* into the cytosol, which in turn triggers cell death. The mPTP, which also exists in yeast, is relevant to cardiac injuries [44] and neurodegeneration [45, 46].

CsA likely protects worm neurons by inhibiting the mPTP. In worm dopaminergic neurons that express α-syn, we propose that there are two inciters of mPTP, and one of these is α-syn. For example, recombinant α-syn binds to purified rat brain mitochondria, which depolarizes the membranes, activates the mPTP, and releases of cytochrome c [47–49]. CsA (1 μM) blocks mPTP activation in human SH-SY5Y cells under conditions in which α-syn accumulates in cells due to inhibition of the proteasome [49]. A similar concentration (~3 μM) of CsA partially blocks neurodegeneration in *C*. *elegans* (Fig 6). The second mPTP inciter, we propose, is low PE or low CL, from aging [50] or modeled herein by RNAi depletion. An issue to address is why the dose-response curves for CsA treated α-syn/*psd-1* depleted worms and α-syn/*crls-1* depleted worms are so similar (Fig 6C). Our view is that mitochondrial depletion of CL or PE in dopaminergic neurons yields identical output: the mPTP is activated. Consequently, CsA inhibits the mPTP with a dose response curve that is independent of whether the instigating event was mitochondrial CL or PE depletion.

**SUL.** SUL is a sulfonamide antibiotic that targets bacterial replication by inhibiting folate biosynthesis. SUL is also a selective inhibitor of the mammalian Cytochrome P450 isozyme CYP2C9 [51], which oxidizes as much as ~15% of drugs undergoing phase I clearance in the liver. CYP2C9 is also expressed in the heart and the brain [52]. SUL was recently identified in a screen of the Prestwick drug library that sought to identify drugs that block light-induced, degenerative loss of photoreceptors that occurs in inherited and age-related retinal degenerative diseases [53]. SUL inhibits light-induced necrosis and apoptosis of mouse-derived photoreceptor 661W cells. It was concluded that SUL blocks this cell death pathway by inhibiting CYP2C9. Since there are potentially at least two targets for SUL, perhaps this explains the bell-shaped dose-response curves (Fig 7C), where at low doses (< 30 μM) SUL partially rescues whereas at high doses (> 30 μM) it is toxic.

A BLAST search query using the human CYP2C9 protein (cytochrome P450 family 2 subfamily C polypeptide 9) sequence against the *C*. *elegans* protein database yielded the Cytochrome P450 cyp-33c11 (6e^-74^). cyp-33c11 is a homolog of human gene CYP2J2. CYP2J2 is conserved in 86 organisms, including chimpanzee, Rhesus monkey, dog, cow, mouse, rat, chicken, zebrafish, fruit fly, mosquito, and frog. In humans, the CYP2J2 and CYP2C9 isozymes carry out the epoxidation of endogenous arachidonic acid in the heart and brain, respectively. If SUL inhibits worm cyp-33c11 the conversion of arachidonic acid from membrane phospholipids to vasoactive epoxyeicosatrienoic acids should be blocked, which should increase the level of arachidonic acid in cells. Arachidonic acid upregulates syntaxin 1 and promotes its interaction with the SNARE complex [54]. Strikingly, α-syn binds to arachidonic acid, which blocks arachidonic acid-induced SNARE interactions, both *in vitro* and *in vivo* [54]. One possibility is that in α-syn/EV/SUL worms, soluble, monomeric α-syn binds and sequesters most of the arachidonic acid molecules, thereby blocking SNARE-mediated exocytosis. In contrast, in α-syn/*psd-1*/SUL worms, aggregated α-syn fails to bind to arachidonic acid; consequently, DA neuron loss is partially rescued compared to α-syn/EV/SUL worms (Fig 7C).

In summary, treating α-syn expressing worms with MFX, CsA, or SUL caused a 200–400% increase in the number of animals with the normal complement of dopaminergic neurons after 7 days of molecule exposure. We hypothesize that neuroprotection respectively observed for each drug comes from MFX scavenging radicals or converting to PC, CsA inhibiting the mPTP, or SUL inhibiting CYP2J2 and sub-family members. Whether these drug candidates, singly or in combination, protect against α-syn-associated pathology in PD patient-derived iPSCs will be the subject of future investigations.

## Materials and Methods

### Yeast Strains, Media, and Materials

The *S*. *cerevisiae* wild-type strain BY4741 (MATa *his3Δ1 leu2Δ0 met15Δ0 ura3Δ0*) and the deletion strain *psd1*Δ (BY4741; *psd1*::*kanMX6*) used in this study were purchased from Open Biosystems. The plasmid used was pAG426–α-syn, where human wild-type α-syn is under the control of the Gal1 promoter [24]. Synthetic complete (SC) dropout media were prepared according to ref. [55]. Cells were transformed with Gal-inducible plasmids using the lithium acetate method, and transformants were pre-grown in SC–Sucrose-uracil (URA) (2% wt/vol) dropout media to maintain the selection for plasmids. α-syn expression was induced in the same dropout media but with 2% (wt/vol) galactose replacing the sucrose. Dropout media was purchased from Sigma-Aldrich and United States Biological, and unless otherwise noted, all other chemicals were purchased from Sigma-Aldrich. Yeast cells in liquid media were grown with shaking at 30°C. The ER and cell wall stress β-galactosidase assays are described in [24].

### High-Throughput Screen

The Prestwick library, containing 1121 number chemically and therapeutically diverse drugs, was screened for compounds that enhanced the growth of *psd1*Δ yeast cells with or without α-syn. These cells were pre-grown in SC–Sucrose-URA (2% w/v) dropout media for approximately 24 h at 30°C with shaking. Cells were pelleted (4000 x g), washed, and re-suspended in SC-URA galactose media to yield OD_600_ nm of 0.4. 135 μL aliquots of yeast cells were pipetted robotically into each well of the 96 well plates. Library compounds diluted in SC-URA galactose (15 μL) were added to each well robotically to yield a final drug concentration of 5 μM. Plates were incubated with gentle shaking for 20–24 h at 30°C in a humidified incubator. Quantitative changes in growth were assayed via optical density changes, whereby *psd1*Δ cells +EV with DMSO (1% v/v), a negative control, would typically have an OD_600 nm_ reading of approximately 0.45, and *psd1*Δ cells + α-syn treated with ethanolamine (5 mM), the positive control, would have an OD_600 nm_ reading of 1.3. The two strongest hit compounds, SUL and MFX, rescued the growth with average z-scores of 17.8 (84% of positive control) and 11.8 (49% of positive control). CsA, a weaker hit, rescued growth with an average z-score of 2.6 (15% of positive control). Compounds were tested in duplicates. The z-factor [56] for the screen was 0.896.

### Dose-Response Test in Yeast

*psd1*Δ cells (+EV or α-syn) were pre-grown in SC–Suc-URA (2% wt/vol) dropout media for approximately 24 h at 30°C with shaking. Cells were pelleted (4000 x g), washed, and resuspended in SC-URA 2% galactose media to yield OD 600 nm of 0.4. 135 μL aliquots of yeast cell suspensions were pipetted robotically into each well of 96-well plates. Several stock solutions of each drug were prepared in DMSO, and typically 15 μL of a stock solution was added per well to yield drug concentrations from 0.005 to 300 μM. Plates were incubated for 24 h at 30°C in a humidified incubator, and the OD was measured using a plate reader.

### RNA Interference (RNAi)

*psd-1 and crls-1* RNAi feeding clones were purchased from Geneservice. Bacteria containing these plasmids were isolated and grown overnight in LB media with 100 μg/ml ampicillin. Nematode growth media plates containing 1 μM IPTG were seeded with RNAi feeding clones and allowed to dry. L4 staged hermaphrodites were transferred to corresponding RNAi plates and allowed to lay eggs overnight to synchronize the F1 progeny. The dopaminergic neurons in the F1 progeny of the RNAi-treated worms were analyzed for neurodegeneration at day 7 following incubation at 20°C. *C*. *elegans* strain UA196 [*sid-1(pk3321)*; P_*dat-1*_::α-syn, P_*dat-1*_::GFP; P_*dat-1*_::*sid-1*, P_*myo-2*_::mCherry] that expresses α-syn, GFP, and SID-1 in the dopaminergic neurons and is susceptible to RNAi selectively in dopaminergic neurons was used in this study [28].

### Dopaminergic Neurodegeneration Analyses in *C*. *elegans*

*C*. *elegans* dopaminergic neurons were analyzed for degeneration as previously described [57]. Strain UA196 was treated with *psd-1*, *crls-1* or EV dsRNA. Nematodes were synchronized, grown at 20°C, and analyzed at day 7 of development for α-syn-induced dopaminergic neurodegeneration. On the day of analysis, the six anterior dopaminergic neurons were examined in 30 adult hermaphrodite worms, in triplicate. These worms were immobilized on glass coverslips using 3 mM levamisole and transferred onto 2% agarose pads on microscope slides. The analysis was carried out using a Nikon E800 with an Endow GFP filter cube (Chroma). Worms were considered normal when all six anterior dopaminergic neurons were present without any signs of degeneration, as previously reported [57]. In total, at least 90 adult worms were analyzed for each RNAi treatment (30 worms/trial; a total of 3 trials). Statistical analyses were performed using two-way ANOVA and a Tukey's or Sikdak's post hoc analysis and are means ± standard deviation (*p* < 0.05) using GraphPad Prism (version 6).

### Pharmacological Treatment of *C*. *elegans*

SUL (Sigma-Aldrich), choline chloride (Avantor), MFX (Sigma-Aldrich) and CsA (Sigma-Aldrich) were dissolved in corresponding solutions as DMSO or water and then added to pre-autoclaved media, with the volume of compound solution taken into account. SUL, MFX and CsA were tested in *C*. *elegans* over a range of concentrations as described in the Results and Figure Legends (0, 1, 2, 3.75, 7.5, 15, 30, 40 μM with 0.1% DMSO in the media). *C*. *elegans* were exposed to a lower concentration range of choline chloride (0, 1.25, 2.5, 5, 10 μM) for consistency with the yeast experiments. All 35 mm worm plates were seeded with 300 μL concentrated HT115 *E*. *coli*. Unless mentioned in the results section, *C*. *elegans* were exposed to drugs from hatching through day 7 of adulthood and analyzed for dopaminergic neurodegeneration.

### *C*. *elegans* Semi-Quantitative RT-PCR Analysis

Sixty worms of the strain UA196 [*sid-1*(pk3321); P_*dat-1*_::α-syn, P_*dat-1*_::GFP; P_*dat-1*_::*sid-1*, P_*myo-2*_:: mCherry] were grown to day 7 at 20°C. They were harvested from drug treatments or solvent control plates (30 μM MFX/0.1% DMSO; 30 μM CsA/0.1% DMSO; 30 μM SUL/0.1% DMSO; 10 μM CC/ddH_2_O). Worms were transferred to fresh drug/RNAi plates as needed to avoid starvation. Total RNAs were extracted from control and drug-treated worms as described previously [58]. Total RNA was quantitated using a Nanodrop and 1 μg of total RNA of each sample was used to synthesize first-strand cDNA using MMLV-RnaseH(-) transcriptase (Promega). Using the cDNA as templates, PCR reactions were conducted with GoTaq polymerase (Promega) at 59°C annealing temperature. Primer sequences are as follows:

α-syn forward primer: GGATGTATTCATGAAGGACTTTCAAAG
α-syn reverse primer: GGCTTCAGGTTCGTAGTCTTG
*ama-1* forward primer: CGAGTCCAACGTACTCTCC
*ama-1* reverse primer: GATGTTGGAGAGTACTGAGC

PCR products were loaded onto a Gel Red (Sigma) stained 0.8% agarose gel. An image was captured by FujiFilm LAS 4000. Band intensities were compared by digital imaging using MetaMorph software. Fluorescent band intensities were normalized using the following equation: α-syn/*ama-1*.

## Supporting Information

S1 Fig Raw data from screen of the Prestwick library. Plots of Z-score versus the drugs in Prestwick plates 1–7 (**A**) and 8–14 (**B**). The Z-score is how many standard deviations a reading (OD value) point is from the mean of all the OD values on a plate. High variability of the samples leads to lower Z-scores. The high Z-scores for SUL and MFX are due to a combination of the high effectiveness of these drugs and the low variability in the screen as a whole.
(TIF)

S1 Table Raw data from Prestwick plates 1–7. This file contains data from the screening of Prestwick plates 1–7. The values are absorbance at 600 nm. The left column (A1-H1) contains the positive control (ethanolamine).
(XLSX)

S2 Table Raw data from Prestwick plates 8–14. This file contains data from the screening of Prestwick plates 8–14. The values are absorbance at 600 nm. The left column (A1-H1) contains the positive control (ethanolamine).
(XLSX)

S3 Table List of hits. This file contains the list of hits (structure, plate name, and plate well).
(XLS)

S4 Table List of Prestwick library drugs. This file contains the information on the library compounds (chemical name, plate name, plate well).
(XLS)
