# Supplementary figures and images for: Chemical Compensation of Mitochondrial Phospholipid Depletion in Yeast and Animal Models of Parkinson’s Disease
Source: PLoS One. 2016 Oct 13;11(10):e0164465. doi: 10.1371/journal.pone.0164465 (PMC5063346; doi:10.1371/journal.pone.0164465)

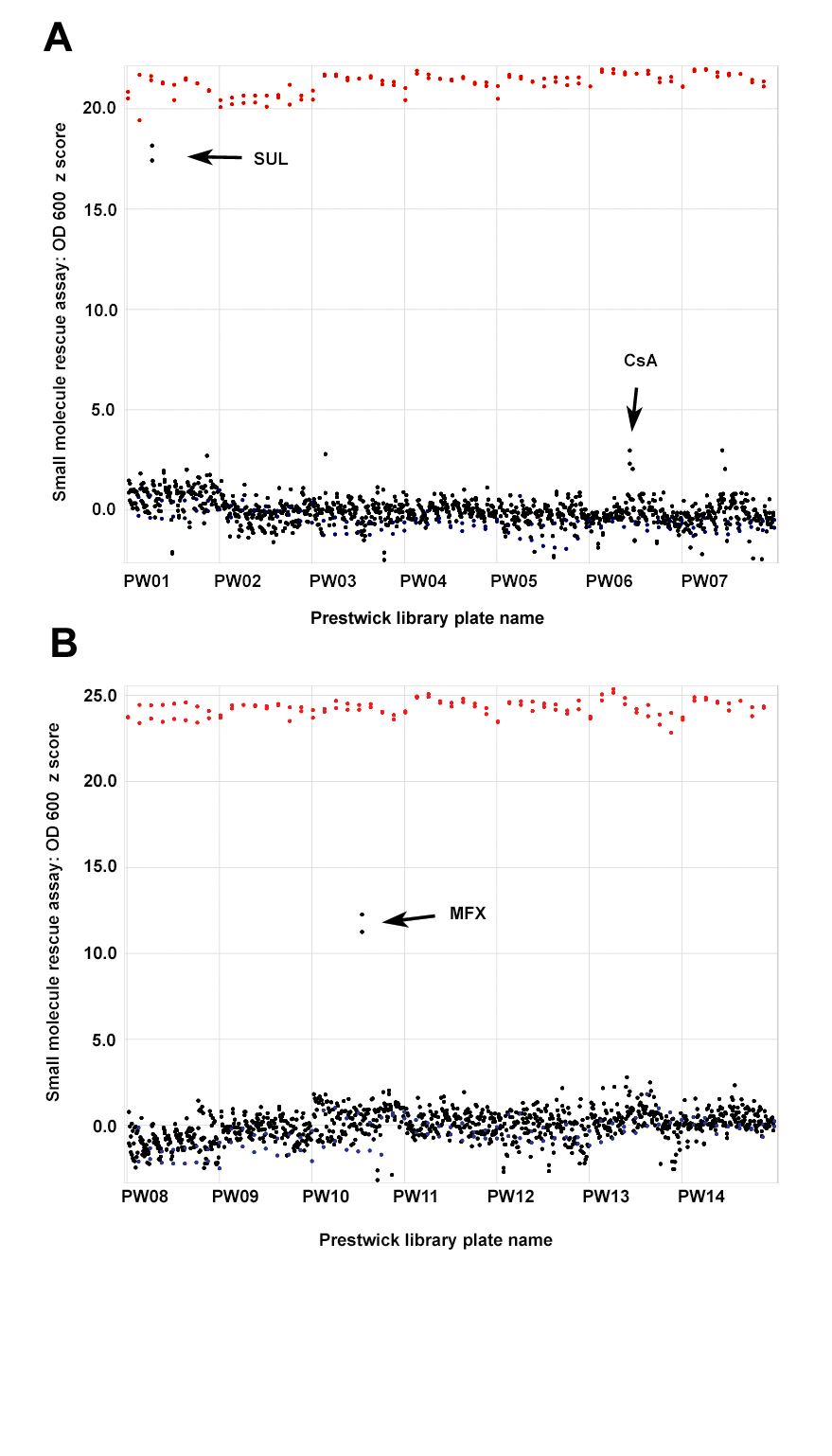

Supplement: S1 Fig — Plots of Z-score versus the drugs in Prestwick plates 1–7 (A) and 8–14 (B). The Z-score is how many standard deviations a reading (OD value) point is from the mean of all the OD values on a plate. High variability of the samples leads to lower Z-scores. The high Z-scores for SUL and MFX are due to a combination of the high effectiveness of these drugs and the low variability in the screen as a whole. (TIF) [file pone.0164465.s001.tif]
